# Natural Killers: Opportunities and Challenges for the Use of Bacteriophages in Microbial Food Safety from the One Health Perspective

**DOI:** 10.3390/foods12030552

**Published:** 2023-01-26

**Authors:** Maria Lavilla, Pilar Domingo-Calap, Sandra Sevilla-Navarro, Amaia Lasagabaster

**Affiliations:** 1 AZTI-BRTA, Food Research, 48160 Derio, Spain; 2 Instituto de Biología Integrativa de Sistemas, Universitat de València-CSIC, 46980 Paterna, Spain; 3 Departamento de Producción y Sanidad Animal, Salud Pública Veterinaria y Ciencia y Tecnología de los Alimentos, Facultad de Veterinaria, University Cardenal Herrera-CEU, 46115 Alfara del Patriarca, Spain

**Keywords:** bacteriophages, food safety, antimicrobial resistance (AMR), *Campylobacter*, *Salmonella*, poultry, *Listeria monocytogenes*, food production, *Vibrio*, aquaculture

## Abstract

Ingestion of food or water contaminated with pathogenic bacteria may cause serious diseases. The One Health approach may help to ensure food safety by anticipating, preventing, detecting, and controlling diseases that spread between animals, humans, and the environment. This concept pays special attention to the increasing spread and dissemination of antibiotic-resistant bacteria, which are considered one of the most important environment-related human and animal health hazards. In this context, the development of innovative, versatile, and effective alternatives to control bacterial infections in order to assure comprehensive food microbial safety is becoming an urgent issue. Bacteriophages (phages), viruses of bacteria, have gained significance in the last years due to the request for new effective antimicrobials for the treatment of bacterial diseases, along with many other applications, including biotechnology and food safety. This manuscript reviews the application of phages in order to prevent food- and water-borne diseases from a One Health perspective. Regarding the necessary decrease in the use of antibiotics, results taken from the literature indicate that phages are also promising tools to help to address this issue. To assist future phage-based real applications, the pending issues and main challenges to be addressed shortly by future studies are also taken into account.

## 1. Introduction

Food and water are the main routes of transmission of more than 200 known infectious diseases, many of which are attributed to bacteria [1]. Among these, the main bacterial foodborne pathogens, in terms of occurrence and seriousness, are *Salmonella enterica*, *Campylobacter* spp., *Escherichia coli*, *Listeria monocytogenes*, *Staphylococcus aureus,* and *Clostridium botulinum*. Considering waterborne diseases, the genus *Vibrio* also enters this important group, besides *E. coli* and *S. enterica* serovar Typhi [2]. Besides the potential serious clinical symptoms, food- and water-borne diseases represent an enormous economic burden for health systems (treatment, potential hospitalization, loss of working days, etc.) and some of them, such us *L. monocytogenes*, can be fatal to humans. Accordingly, measures for the prevention of their presence and proliferation in food products should be comprehensive and strict.

On the other hand, food production is a complex and multifaceted procedure which starts from the growth of animals and the harvest of plants through different practices up to the point of their consumption by customer. Along this path, there are many chances for bacterial contamination. Many of those pathogenic bacteria are considered as ubiquitous and normal microbiota in the environment and in animals. They may cause infections as zoonotic pathogens, usually infecting humans through cross-contamination, for instance, during improper food handling and preparation, especially if the food products are stored under poor conditions [3,4,5].

**Figure 1:**
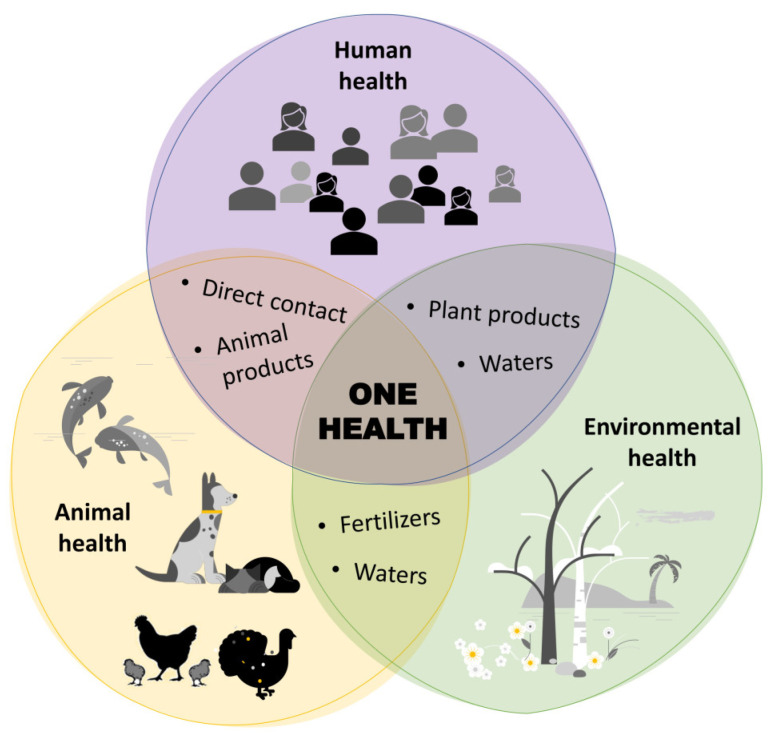
Interconnection between humans, animals, and the environment within the One Health concept.

To reduce the risk, several control measures may be taken to preserve food from contamination with dangerous microorganisms and to reduce foodborne diseases, accordingly. In this sense, the One Health approach is promoted by global organizations for the health of people, animals, plants, and the environment [6]. This promotes a transformation of the agrifood system, which involves many factors, such as sustainable agriculture; animal, plant, forest, and aquaculture health; antimicrobial resistance (AMR); and, of course, food safety [6]. In agreement with this definition, the One Health approach (Figure 1) also ensures food safety by anticipating, preventing, detecting, and controlling pathogens that spread between animals and humans, with special attention paid to AMR bacteria. Indeed, AMR is considered one of the most important environment-related global threats, expected to be the leading cause of human mortality by 2050 [7,8].

Considering the emergence of multidrug-resistant (MDR) bacteria, the development of innovative and effective alternatives to control bacterial infections is becoming an urgent issue. In this scenario, bacteriophages or phages, which are viruses that infect bacteria and the most abundant and diverse biological entities worldwide, are currently gaining an important prominence in the request of new effective alternatives to treat bacterial diseases in humans, animals, and plants [9,10]. Moreover, this “phage biocontrol”, targeting specific pathogenic bacteria, is also increasingly accepted as a natural, effective, and inexpensive food (and feed) safety strategy [11,12]. 

**Figure 2:**
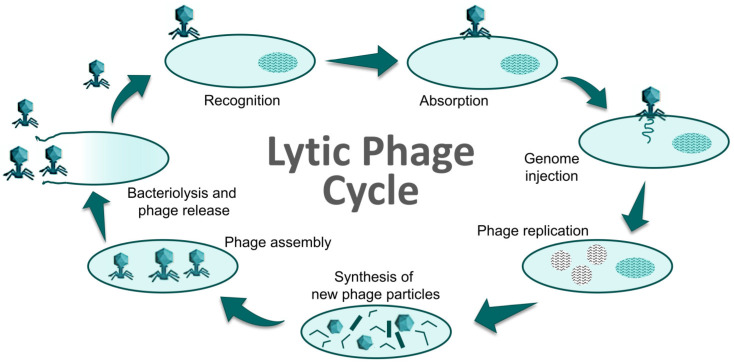
Cycle of a lytic phage resulting in cell death (bacteriolysis). Adapted from [20].

Phages are usually classified according to the strategies they use to escape their hosts, into lysogenic (temperate) phages and lytic (virulent) phages [13,14]. Lytic phages cause the death (lysis) of their host at the end of their lytic cycle (Figure 2) and, consequently, these are the most suitable phages to be used in biocontrol applications [15]. Because of their high specificity, phages eliminate target bacteria without affecting the normal and beneficial microbiota of the host, the food, or the environment. Moreover, as they are already widely present in the environment, phages are not supposed to have any harmful consequence on the quality of food or animal and human health [16]. The safety and effectiveness of phage-based biocontrollers is reflected in the fact that several preparations have been approved for use in food [17]. In addition, they can be used alone or in combination with other phages (phage cocktails) in order to achieve a broader host range, or with antibiotics or disinfectants to control bacterial infections more effectively [18]. In fact, phage-antibiotic synergy (knowns as the PAS effect), has been observed in some phage–antibiotic combinations [19]. 

Phages are potent antimicrobial agents against most pathogenic bacteria, and can be usefully implemented for their environmentally-friendly application at each stage of the farm-to-fork continuum. This corresponds to the One Health concept for reducing foodborne diseases: they can be used in many applications, such as phage-therapy in animal production, cleaning of the livestock, disinfection and sanitation of equipment and contact surfaces on farms and in industry, biocontrols in fresh meats and produce, and also as natural preservatives to extend product shelf-life [1,21,22].

Nevertheless, many factors, such as the target bacteria, the application route, the phage administration timing (prophylactic vs. therapeutic), the number of phage administrations (single vs. repeated), the number of phages used (single vs. cocktail), food composition, or the storage temperature, for instance, are factors to be taken into account, as they may imply differences in the results regarding the effects of phages on the biocontrol of pathogens in foods and animals [15,23].

## 2. Phage Biocontrol in Animal Husbandry for Food Production

The raise of MDR bacteria causing dissemination of AMR requires alternative strategies to combat pathogenic bacteria. Phages possess some unique characteristics, such as high species-specific nature, relatively easy handling, self-replication, and self-limiting [24,25], that make them a promising alternative for effectively inhibiting the colonization of pathogenic bacteria and reducing animal and zoonotic diseases [17,26,27]. 

### 2.1. Control of Campylobacter and Salmonella in Broilers

*Campylobacter* and *Salmonella* are widely distributed in most warm-blooded animals, such as poultry, and contaminate foods during slaughter, handling, and/or carcass processing. The main source of human infection for both bacteria is the consumption of contaminated products of animal origin, mainly undercooked eggs and poultry meat [28]. Due to their importance in public health, both bacteria are controlled by the European Community legislation. However, despite efforts to control them, new foodborne outbreaks (FBO) of salmonellosis and campylobacteriosis emerge every year [28,29].

*Campylobacter* is the most common foodborne pathogen causing zoonotic illnesses in humans. The majority of campylobacteriosis cases are caused by *Campylobacter jejuni* (∼89%) and *Campylobacter coli* (∼10%), with only a few cases (<1%) associated with *C. fetus*, *C. upsaliensis,* and *C. lari* [28]. Although treatment is not generally required, quinolones, macrolides, and tetracyclines are antibiotics used to combat severe infections [30,31]. The alarming emergence of *Campylobacter* resistance to these drugs compromises the effectiveness of therapeutic treatments and lead the World Health Organization (WHO) to include *Campylobacter* in its global priority list of antibiotic-resistant pathogens [32].

Poultry is considered the natural primary reservoir of *Campylobacter* spp., with *C. jejuni* being the predominant species colonizing broiler chickens. Colonization naturally occurs by horizontal transmission from the environment, and the infection rapidly spreads within the flock, reaching more than 10^7^ CFU/g in their intestinal tract before slaughter [33]. The prevention and control of *Campylobacter* in poultry is a food safety issue of high priority, since it is widely accepted as a significant risk factor of human campylobacteriosis. Reducing the *Campylobacter* load in broiler intestines by 3 logs prior to slaughter was estimated to reduce the risk of human campylobacteriosis attributable to the consumption of poultry meat by 58% [34].

Even though no phage-based products with specific activity against *Campylobacter* are commercially available yet, phage biocontrol is one of the most promising alternatives under development to address the reduction in this pathogen in the poultry reservoir [35]. *Campylobacter*-infecting phages (also called campylophages) have been isolated wherever their hosts are present, including in both poultry environmental samples and food products [33,36]. Campylophages have been classified into three groups (groups I, II, and III) according to their genome size [37]. Group I phages (320 kb) have rarely been isolated, whereas phages of group II (180 kb; Cp220virus) and group III (140 kb; Cp8virus) are common and contact their target hosts via flagella and capsule polysaccharide (CPS) receptors, respectively [33]. 

Different studies have reported the use of campylophages to reduce *Campylobacter* counts in the gastrointestinal tracts of broiler chickens (Table 1), without affecting their health and well-being [38] or their gut microbiome [39]. These studies have shown reductions of up to 5 log in the cecal counts of *Campylobacter* colonized chickens, including AMR *Campylobacter* strains [38]. As mentioned initially, the high variability reported in *Campylobacter* reduction might be dependent on a number of factors, such as the susceptibility of each strain to the applied phages [40,41,42,43], the route of phage administration [41,42], the dose and timing of application [38,39,40,42,44,45] or the development of phage-resistant *Campylobacter* mutants [40,41]. The use of polyphage therapy (campylophage cocktails) instead of single phages has been also studied for a broader host range [39,41,42,44]. Unfortunately, achieving complete *Campylobacter* elimination in broilers may be a difficult task. Nevertheless, the careful design and application of campylophage cocktails targeting different cell receptors (containing both group II and group III campylophages) has been suggested as the best approach to successfully combat *Campylobacter,* resulting in an efficient reduction in *Campylobacter* at the farm level, with a significant impact on food safety and public health [39,46].

*Salmonella* is the second zoonotic pathogen responsible for human gastrointestinal diseases. In fact, millions of human salmonellosis cases are reported worldwide every year, resulting in thousands of deaths. In the United States, *Salmonella* causes around 1.2 million cases every year, of which there are around 23,000 hospitalizations and 450 deaths [47]. In Europe, a total of 60,050 confirmed cases in humans were reported in 2021 by the European Surveillance System [28], reporting an increase of 14.3% in comparison with the previous year. Different serovars have been associated with salmonellosis, yet *Salmonella* Enteritidis, followed by *Salmonella* Typhimurium, has been the most common serovar related to FBO in humans worldwide [48], as well as in the EU [28].

**Table 1:** Examples of the use of bacteriophages for reducing the incidence of *Campylobacter* spp. and *Salmonella* spp. in broilers (pre-harvest stages of production).

Animal (Age)	Bacteria Load ^1,2^	Phage	Application Method and Dose ^3^	Bacterial Reduction	Ref.
***Campylobacter* spp.**					
Chickens (38 days old)	*C. jejuni* AMR * 10^8^ (^1^)	ϕ16-izsam ϕ7-izsam	Oral (37 dpi); single dose; sequential application (24 h) 1 day before slaughtering. 10^7^ 10^8^	1 log reduction 2 log reduction	[38]
Chickens (24 days old)	*C. jejuni* HPC5 10^8^ (^1^)	Cocktail (2): CP20 ^GII^ CP30A ^GIII^	Oral (4 dpi); single dose; 10^7^	2.4 and 1.3 log reduction after 2 and 5 dpt	[39]
Chickens (25 days old)	*C. jejuni* HPC5 10^7^ (^1^) *C. jejuni* GIIC8 10^7^ (^1^)	CP34 ^GIII^ CP8 ^GIII^ CP8 ^GIII^	Oral (5 dpi); single dose; 10^5^–10^7^–10^9^	0.5–4 log reduction Marginal reductions Initial 5 log reduction and 2 log reduction after 5 dpt	[40]
Chickens (38 days old)	*C. jejuni* 2140CD1 10^7^ (^1^) *C. coli* A11 10^6^ (^1^)	Cocktail (3): ϕCcoIBB35 ^GII^ ϕCcoIBB37 ^GII^ ϕCcoIBB12 ^GII^	Oral (7 dpi); single dose; 10^6^ In feed (7dpi); single dose; 10^7^	1.2 and 1.7 log reduction after 2 and 7 dpt 2 log reduction after 2 and 7 dpt	[41]
3 field trials Chickens (36 days old)	*C. jejuni* 10^2^–10^7^ (^1^)	Cocktail (4): NCTC12672 ^GIII^ NCTC12673 ^GIII^ NCTC12674 ^GIII^ NCTC12678 ^GIII^	Drinking water (7 dpi); single dose; 10^7^	Up to 3.2 log reduction in one field trial No reduction in two field trials	[42]
Chickens (47 days old)	Naturally colonized chickens	Cocktail (4): PH5, PH8, PH11, PH13	Oral; single dose; 10^7^	1.3 log reduction after 1 dpt	[43]
Chickens (10 days old) Chickens (32 days old)	*C. jejuni* C356 10^8^–10^9^ (^1^)	NCTC12671 ^GIII^ Cocktail (2): NCTC12671 ^GIII^ NCTC12669 ^GIII^	Oral (5dpi); 5 doses (24 h interval); 10^10^–10^11^ Oral (7dpi); 4 doses (24 h interval); 10^10^–10^11^	Initial 3 log reduction followed by 1 log reduction over 20 dpt Initial 1.5 log reduction followed by 1 log reduction over 20 dpt	[44]
Chickens (25 days old)	*C. jejuni* HPC5 10^7^ (^1^) *C. coli* OR12 10^9^ (^1^)	CP220 ^GII^	Oral (5 dpi); single dose; 10^7^ 10^9^	2 log reduction 2 log reduction	[45]
Chickens (27 days old)	*C. jejuni* 3871 10^9^ (^1^)	CP14 ^GIII^ Cocktail (2): CP14 ^GIII^ CP81 ^GIII^ CP14 ^GIII^ CP68 ^GII^	Oral (7 dpi); single dose; 5 × 10^8^ Oral (7 dpi); single dose; 5 × 10^8^ Oral (7 dpi); single dose; sequential application (24 h); 5 × 10^8^–5 × 10^10^	1 log reduction after 3 dpt No reduction 3 log reduction after 3 dpt	[46]
***Salmonella* spp.**					
Layer hens (6 weeks old)	*S.* Gallinarum KVCC BA00722 10^8^ (^2^)	ST4 L13 SG3	Feed additive 10^8^ 7 days before and 21 dpi	50% reduction in liver and spleen after 7 dpi; 70% survival rate 75% and 50% reduction in liver and spleen after 7 and 14 dpi, respectively; 75% survival rate 25% and 50% reduction in liver and spleen after 7 and 14 dpi, respectively; 50% survival rate	[49]
Chickens (36 days old)	*S.* Enteritidis P125109 *S.* Typhimurium 4/74 10^8^ (^2^)	Phage cocktail: ϕ151, ϕ25 ϕ10	Oral; single dose 10^11^	1.53 log and 3.48 log reduction of *S*. Enteritidis and *S*. Typhimurium, respectively	[50]
Chickens (6-10 days) Chickens (31-35 days)	*S.* Enteritidis (PT4) 6 × 10^6^ (^2^)	Phage cocktail: CNPSA1 CNPSA3 CNPSA4	Early treatment after challenge: drinking water for 5 consecutive days (from 6 to 10 dpi) Later treatment after challenge: drinking water for 5 consecutive days (from 31 to 35 dpi) 10^9^	1.08 log reduction after later treatment	[51]
Chickens (1 day old) (6 days old)	*S.* Enteritidis 10^3^ (^2^)	Single phage or cocktail: CB4ϕ WT45ϕ	Cloacal drop 1 h pi: WT45ϕ: 10^9^ Oral delivery 1 h pi: WT45ϕ: 10^8^ CB4ϕ: 10^8^ Cocktail: 10^8^	Reduction in *Salmonella* detection to 36% Reduction in *Salmonella* detection to 70%, 65%, and 45% after 1 dpt No significant differences after 48 h	[52]
Chickens (10 days old)	*S.* Enteritidis 10^5^ (^2^)	Phage cocktail	Coarse spray or drinking water 10^8^	Reduction in *Salmonella* detection to 72.7% by aerosol-spray	[53]
Layer hens (40 weeks old)	*S.* Enteritidis (SE^NAR^) 10^8^ (^2^)	Phage cocktail: SP-1 STP-1	Feed additive: 0.2% of the phage cocktail	0.9, 0.57, and 0.38 log reduction in cecum, liver, and spleen at 7 dpt 0.86 log reduction in cecum at 6 dpt	[54]
Layer hens (60 weeks old)	Natural infection	Autophage (AP) Wild-type phage	Spray applications 10^8^ Two single applications in 24 h intervals	1.78 log reduction in feces samples Total elimination of *Salmonella* from the environment	[55]
Chickens (1 to 35 days)	*S.* Enteritidis 10^4^ (^2^)	Bafasal (4 phages cocktail)	Feed additive daily 10^6^	1 log reduction at day 35 of study	[56]

^1^ Cecal/fecal content (CFU/g); ^2^ bacterial oral infection dose (CFU/animal); ^3^ administered phage dose (PFU/mL); dpt: days post-treatment; dpi: days post-infection; pi: post-infection; * AMR: antimicrobial resistant strain.

Although the use of antibiotics has been limited to therapeutics in Europe since 2012, the presence of resistant strains is being considered as a human and veterinary health concern. For instance, the data published by the European Food Safety Authority (EFSA) and the European Centre for Disease Prevention and Control (EDCD) in 2022 reported resistance of *Salmonella* against sulfonamides/sulfamethoxazole (30.1%), tetracyclines (31.2%), and ampicillin (29.8%) [57]. Moreover, resistance to ciprofloxacin was reported in 14.1% of the isolates, which was a slight increase compared with the previous report. Resistance to cefotaxime or ceftazidime was observed to be generally very low (<1%) among *Salmonella* spp. of the isolates. These antimicrobials represent the most important antimicrobial classes (fluoroquinolones and third-generation cephalosporins) used for the treatment of salmonellosis, and they have been classified by the WHO as the highest priority [58]. 

The current situation has encouraged the search for new alternatives, such as the use of phages against *Salmonella* [59]. Several studies showed phage biocontrol success in the poultry sector (Table 1), as it has been shown that phages reduced side effects compared to traditional antibiotic treatments due to their specificity [60]. At the field level, different publications have demonstrated the efficacy of phages in reducing *Salmonella* concentration in chickens. Zbikowska et al. infected chickens with *Salmonella* Gallinarum, and then animals were fed with a cocktail of phages, leading to a significant decrease in *Salmonella* in the organs as well as in the mortality of the chickens [61]. Similar results have been previously reported, showing that phages are a promising tool and an effective alternative to antibiotics [49,62]. Further, reductions of 1.53 log and 3.48 log of *S.* Enteritidis and *S.* Typhimurium, respectively, were reached after the application of a single dose of a phage cocktail [50]. Likewise, statistically significant differences in *S.* Enteritidis reduction after a later phage treatment demonstrated that the application of phages at late stages of broiler growth may be a promising measure for the control of this bacterium in future stages of the production chain [51].

### 2.2. Control of Listeria Monocytogenes in Animals 

*L. monocytogenes* is a well-known pathogen responsible for listeriosis, one of the most serious food- and feed-borne zoonotic diseases worldwide. This pathogen can reach food products by contaminated raw materials or by cross-contamination during different steps of food processing [63,64]. Listeriosis in domestic animals is usually transmitted through the ingestion of contaminated feed and/or pet food, although it can also be transmitted through the upper respiratory tract mucosa, conjunctiva, and wounds [65]. Animal listeriosis is generally associated with encephalitis, abortion, septicemia, and mastitis in ruminants, but also in swine, horses, birds, rodents, fishes, and crustaceans, although an even wider range of animal species can also be affected. 

Since animals act a reservoir and a main source of *L. monocytogenes* to humans, using the One Health concept, it would be reasonable to treat animals to control the introduction of *Listeria* into the food chain. However, the absence of harmonized regulations regarding the presence of *L. monocytogenes* at primary production has led to the low quantity of reported data [28]. Accordingly, as far as we know, there is still a lack of investigation into the use of phages for the control of *L. monocytogenes* in animals, and few examples have been found in the literature. The use of phage P100 has been proposed for the treatment of animals (including humans) infected with *L. monocytogenes* [66], but no further publications have been found regarding the application conditions or phage effectivity. Only one recent publication has demonstrated the potential therapeutic effect of phage LP8 against listeriosis in mice and the feasibility of a combined therapy to reduce the use of antibiotics in animals [67]. As more published research continues to focus on the application of *Listeria* phages in foods and food processing environments, this topic will be discussed in the sections below.

### 2.3. Control of Vibrio spp. in Aquaculture

Water also represents one of the most important methods of dissemination of AMR. Pathogenic microorganisms, such as *Vibrio* spp., occur naturally in water [68] and are the most important environmental human pathogen from aquatic and marine habitats [69,70]. In animals, vibriosis is responsible for important economic losses in turbot, salmonids, sea bass, and shrimps [71,72]. The incidence of all of these infections is rising, favored by the rising of sea water temperature due to climate change [68,73,74]. Additionally, as the global aquaculture production is increasing and is progressively growing into an intensive industry, the concentration of fishes in larger farms also may cause an increase in bacterial disease occurrence [71,73]. 

Although control and hygiene measures are important hurdles to the occurrence of an outbreak, antibiotics are still the most effective chemical agents for controlling *Vibrio* spp. Their abuse has caused the emergence of multidrug-resistant strains, and many *Vibrios* have already become highly resistant to most commercially available antibiotics [75,76,77]. With only a few antibiotics approved for aquaculture, this food source industry is continuously facing the threat of bacterial contamination. Furthermore, to mitigate antibiotic-resistant microorganisms, many countries have introduced strict antibiotic-handling programs, which include proper dosage of antibiotic treatment and objectives such as a 50% reduction in the use of antibiotics by 2030 in aquaculture [78]. Accordingly, the development of alternative biocontrol agents against *Vibrio* for aquatic hatcheries is also an urgent need, especially where vaccines cannot be applied. Some studies have shown the applicability of phages to reduce human pathogenic *Vibrio* spp. from aquaculture (Table 2). 

These studies have demonstrated the potential of phages controlling *V. parahaemolyticus* in vivo. For instance, the VP10 phage cocktail significantly reduced *V. parahaemolyticus* to undetectable numbers in mussels [79], and pVp-1 reduced bacterial growth by five orders of magnitude when phages were added into oysters’ tanks [80]. Additionally, the VPG01 phage remarkably reduced the presence of *V. parahaemolyticus* in artificial seawater and in the aquatic crustacean *Artemia franciscana* [81]. 

For the last two decades, phages have been also studied for controlling animal vibriosis (Table 2), most of them specifically targeting the fish pathogen *Vibrio harveyi*. It has been demonstrated that phages can reduce the mortality of infected shrimp larvae, from 75% (without phages) up to 20% [82]. More recently, a phage named Virtus has shown an important protective effect against mortality caused by *V. harveyi* on seabream larvae [83]. Phages have been also demonstrated to be useful weapons against *V. anguillarum* infections. The application of the phage CHOED increased *Salmo salar* survival rates in aquaculture conditions from 60% to 100% [84]. A similar result, namely a mortality rate less than 3%, was achieved using the VP-2 phage on zebrafish larvae as an infection model [85]. 

In aquaculture, phages may have additional advantages: Their specificity allows them to kill the target pathogenic *Vibrio* spp., while being unable to kill beneficial *Vibrio* spp. in fish microbiota. In addition, phages are especially easy to administer in water, and have the benefit of treating both the farm environment (water and facilities) and the farmed species [88]. This evidence suggest that phage therapy could be a viable alternative to protect and treat fish against these bacteria in different developmental stages, as well as preventing water-borne human *Vibrio* infections. 

## 3. Phage Biocontrol at the Post-Harvest and Post-Slaughtering Stage

Pathogenic bacteria mostly contaminate the food products during the steps of harvesting, slaughtering, processing, and packing, and are becoming resistant to available antibiotics. Due to their potential, at present, there are many studies on post-harvest phage biocontrol interventions (direct food applications) for *L. monocytogenes*, *Salmonella* spp., *C. jejuni*, *Vibrio* spp., *E. coli* O157:H7, *Cronobacter sakazakii*, *Shigella* spp., and *Staphylococcus aureus* [89,90,91,92,93], among others. Below, we review some studies on the effectiveness of bacteriophage biocontrol of the selected foodborne pathogens on different food products.

### 3.1. Campylobacter

The consumption of contaminated raw and undercooked poultry meat is the major source of human campylobacteriosis [28]. The application of specific phages has been explored as a pre-harvest strategy to reduce *Campylobacter* colonization in broilers, as mentioned previously. Furthermore, although no commercial phage preparation is currently available for the biocontrol of *Campylobacter* in foods, some studies have also reported the efficacy of campylophages as a post-slaughter biocontrol strategy to reduce *Campylobacter* counts in different poultry products (Table 3) without affecting the remaining microbiota [33]. Three studies found a reduction of around one log in *C. jejuni* loads when artificially contaminated chicken skin samples were treated with phages and stored in refrigerated conditions (4–5 °C) [26,94,95]. The use of a high multiplicity of infection (MOI) of 10^3^ has been suggested as the best approach to reduce *Campylobacter* load with no development of phage-resistant *Campylobacter* mutants [26]. 

The combination of phage treatment and freezing was shown to cause a further *Campylobacter* reduction of up to 2.5 log in chicken skin [26]. Under refrigerated temperatures, phage treatment was effective as a function of the campylophage receptor [95]: group II campylophages, which reversibly bind to host flagella, resulted to be unsuitable for *Campylobacter* biocontrol. Therefore, although the application of cocktails including both group II and III campylophages has been suggested to reduce *Campylobacter* colonization in broilers (Section 2.1), the use of only group III campylophage cocktails was proposed to successfully combat *Campylobacter* through post-harvest application [95]. While some authors reported negligible *Campylobacter* reduction in contaminated chicken meat [96], other researchers achieved a reduction of more than 1.5 log in raw and cooked beef [97] and chicken meat [98] after refrigerated storage for 1 and 2 days, respectively. 

The ability of campylophages to reduce *Campylobacter* counts from chicken carcasses or food products may represent a promising approach to eliminating the risk of contamination from a finished product. Furthermore, the application of *Campylobacter*-specific phages could also provide an innovative alternative for surface sanitizing to reduce biofilms on food contact surfaces [33,99]. 

**Table 3:** Examples of the effectiveness of phage biocontrol of target foodborne pathogens on different food products.

Food	Bacteria Load ^1^	Phage	Application MOI * and Method	Result/Bacterial Reduction	Ref.
*Campylobacter* spp.					
Chicken skin	*C. jejuni* PT14 4 and 6 log	ϕ2: NCTC12674 ^GIII^	MOI: 0.01–1 MOI: 10–10^3^ spread on surface	Negligible reduction 1 log reduction after 30 min, 3 d and 5 d at 4 °C 2.5 log reduction after 5 d at −20 °C	[26]
Chicken skin	*C. jejuni* C222 4 log	NCTC12673 ^GIII^	MOI: 10^2^ spread on surface	1 log reduction after 1 d at 4 °C	[94]
Chicken neck skin	*C. jejuni* NCTC12662 4 log	F356^GIII^ F357 ^GIII^ F379 ^GIII^ Cocktail (2): F356 ^GIII^ F357 ^GIII^	MOI: 10^3^ spread on surface	0.5 log reduction at 5 °C 0.5 log reduction at 5 °C Negligible reduction at 5 °C 0.7 log reduction after 1 d at 5 °C	[95]
Chicken meat	*C. coli* NCTC 126683 *C. jejuni* NCTC 11168 3 log	NCTC12684 ^GII^ CP81 ^GIII^	MOI: 10^4^ spread on surface	No reduction at 4 °C No reduction at 4 °C	[96]
Raw and cooked beef	*C. jejuni* 4 log	Cj6	MOI: 10^4^ spread on surface	1.5 and 2 log reduction after 1 d at 5 °C in raw and cooked beef, respectively	[97]
Chicken meat	*C. jejuni* 4 log	CJ01	MOI: 10^2^ spread on surface	1.7 log reduction after 2 d at 4 °C	[98]
***Salmonella* spp.**					
Commercial broiler and turkey carcasses	*S.* Enteritidis (PT 13A) 20 CFU *S.* Enteritidis (PT 13A) 31 CFU *S.* Enteritidis host *S.* Enteritidis field (S9, S14)	PHL 4 72 wild-type phages	Broiler carcasses: MOI: 10^4^ to 10^10^ spray Turkey carcasses: MOI: 10^6^ to 10^8^ rinsed	50–100% reduction 60% reduction	[100]
Breast and eggs	*S.* Enteritidis LK5, UA1894 Breast: 10^6^ Eggs: 10^7^	UAB_Phi 20 UAB_Phi78 UAB_Phi87	10^9^ PFU (MOI: 10^3^) rinse 10^10^ (MOI:10^3^) spray	2.0 log reduction 0.9 log reduction	[101]
Liquid eggs and chicken meat	*S.* Enteritidis Liquid eggs: 10^4^ Chicken meat: 10^5^	SE07	10^11^ (MOI 10^7^) Direct addition of 100 mL 10^12^ (MOI 10^7^) spray	2 log reduction after 12, 24, and 48 h 2 log reduction after 12, 24, and 48 h	[102]
Breast samples	*S.* Enteritidis ATCC13076 CVCC2184 4 × 10^5^	PA13076 PC2184	Single phage: 4 × 10^9^ (MOI: 10^4^) Cocktail: 4 × 10^9^ (MOI: 10^4^)	2 log reduction Phage PC2184 better than phage PA13076 at 4 °C and 25 °C 2 log reduction	[103]
Chicken breast	*S.* Typhimurium ATCC 14,028 *S.* Enteritidis ATCC 4931 *S.* Heidelberg ATCC 8326 3 logs	SalmoFresh ^TM^ (6 phages)	MOI: 10^6^ spray	0.7 and 0.9 log reduction on day 0 and 1, at 4 °C 1 log reduction on day 7 with modified atmosphere at 4 °C 0.8, 0.9, and 0.4 log reduction at 0, 4, and 8 h at room temperature, respectively	[104]
Chicken and turkey meat	*S.* Enteritidis ATCC 13,076 *S.* Typhimurium ATCC 6539 *S.* Heidelberg ATCC 8326 1.5 × 10^3^ 1.25 × 10^3^	SalmoLyse^®^	2 × 10^6^, 4 × 10^6^, 9 × 10^6^ MOI: 2 × 10^3^, 3 × 10^3^, 6 × 10^3^ spray 9 × 10^6^ and 2 × 10^7^ MOI: 7 × 10^3^, 1 × 10^4^ spray	60%, 71%, and 88% reduction from chicken meat at 2 × 10^6^, 4 × 10^6^, 9 × 10^6^ PFU/mL, respectively 68% and 86% reduction from turkey meat at 10^6^ and 10^7^ PFU/g, respectively	[105]
Chicken meat	*S.* Typhimurium JCW-3001 *S.* Enteritidis VDL-133 *S.* Dublin SP-598 5 log	SalmoFREE^®^ (6 phages)	10^8^, 10^9^ (MOI: 10^3^, 10^4^) immersion	1.9–2.0 log reduction in combination with plant-based essential oils	[106]
Chicken meat	*S.* Enteritidis 10^4^	PhageGuard S^®^ (2 phages)	10^7^ (MOI: 10^3^) immersion	1.5 log reduction after 24 h	[107]
***Listeria monocytogenes***					
Raw salmon	4 log 2 log	Listex™ P100	MOI: 1, 10, 10^2^, 10^3^, 10^4^ spread on surface MOI: 10^6^ spread on surface	Marginal reductions at lower MOIs, but up to 3 log reduction at higher MOIs 1.4 log reduction (1 d) No regrowth after 10 d at 4 °C	[108]
Raw hake Raw salmon Smoked salmon	3 log	Listex™ P100	Automated spray MOI: 10^4^	1.2 and 2.0 log reduction after 1 d and 7 d at 4°C (hake) 0.8 and 1.0 log reduction after 1 d and 7 d at 4°C (raw salmon) 0.8 and 1.6 log reduction after 1 d and 30 d at 4°C (smoked salmon)	[109]
Smoked salmon	3 log	ListShield™ (6 phages)	MOI: 10^3^ spray	0.4 and 1 log reduction	[110]
RTE chicken breast roll	2, 4, and 5 log	FWLLm1	MOI: 10^5^, 10^3^, 10^2^ spread on surface	Rapid 1.5–2.5 log at 5–30 °C. Regrowth prevented over 21 d at higher MOI and 5 °C (vacuum packed)	[111]
Cooked turkey and roast beef	3 log	Listex™ P100	MOI:10^4^ spread on surface	1.7 log and 2.1 log, respectively, after 28 d at 4 °C	[112]
Sliced cooked ham	4 log	Listex™ P100	MOI: 10^4^ spread on surface	Rapid 1 log reduction 2 log reduction after 28 d at 4 °C	[113]
Dry-cured ham	2, 3, 4 log	Listex™ P100	MOI: 10^2^–10^6^ spread on surface	2.5 log to undetectable (highest MOI) after 14 d at 4 °C	[114]
Milk	5 log	Monophages LMP1 and LMP7	MOI:10 addition to milk	0.5–3.3 log at 4 °C	[115]
“Queso fresco” cheese	4 log	Listex™ P100	MOI: 10^4^ spread on surface	2 log reduction	[116]
Soft cheeses	3 log 1, 2 log	A511	MOI: 10^5^ in the smearing solution MOI: 10^6^, 10^7^	2.5–3 log reduction during the 21 d ripening period >6 log reduction (below the limit of detection)	[117]
Hard cheese	4 log	ListShield™ (6 phages)	MOI: 10^4^ spray	0.7 log reduction	[110]
Lettuce Apple slices	3 log 4 log	ListShield™ (6 phages)	MOI: 10^4^, 10^5^ spray MOI: 10^2^ spray	1.1 log reduction at higher MOI 1 log reduction	[110]
Fresh-cut apple and melon	5.5 log	Cocktail (12 phages) LM-103 Cocktail (6 phages) LMP-102	MOI: 10^2^ spray	Below 0.4 log reduction in apple 2.0–4.6 log reduction in melon	[118]
Fresh-cut apple, pear, and melon slices. Apple, pear, and melon juices	5 log 5 log	Listex™ P100	MOI: 10^3^ spread on surface MOI: 10^3^ addition to juice	None, 0.6, and 1.5 log reduction in apple, pear, and melon slices after 8 d at 10 °C None, 2, and 8 log reduction in apple, pear, and melon juices after 8 d at 10 °C	[119]
Celery and enoki mushroom	5 log	Mix of 3 phages: LMPC01 LMPC02 LMPC03	MOI: 10	2.2 and 1.8 log reduction in celery and enoki mushroom after 7 d at 4 °C	[120]
***Vibrio*** **spp.**					
Oysters *Crassostrea gigas*	*V. parahaemolyticus* CRS 09-17, AMR* 1.6 × 10^6^ CFU in each oyster	pVp-1	2 × 10^7^ PFU/oyster (MOI: 10) surface of flesh	6 log CFU/mL growth reduction after 12 h	[80]
Fresh fish flesh	*V. parahaemolyticus* FORC_023 3 × 10^4^	VPG01	MOI: 1 MOI: 10 surface direct application	1 log reduction (MOI: 1) Counts under the detection limit after 6 h (MOI: 10)	[81]
Cutting board	*V. parahaemolyticus* FORC_023 3 × 10^4^ CFU/cm^2^	VPG01	MOI: 10^3^ surface direct application	3 log reduction in utensil surface	[81]
Raw fish flesh slices	*V. parahaemolyticus FORC_023* 3 × 10^4^	VPT02	MOI of 0, 1, or 10 surface direct application	2 log reduction after 6 h at 25 °C (MOI: 10)	[121]
Shrimp	*V. parahaemolyticus* F23	F23s1 Recombinant endolysin ORF52	MOI: 10^3^ in vitro 20 µmol/L	Growth inhibition at 25 °C for 12 h Decreased OD_600_ after 60 min The endolysin also showed lytic activity against a panel of 23 drug-resistant *V. parahaemolyticus*	[122]
Manila clams	*V. parahaemolyticus* Vp-KF4 1 × 10^4^	Vpp2	MOI of 1, 10, or 100	2.1 log reduction at 25 °C until 24 h No effect of treatment at 4 °C	[123]
Oysters	*V. parahaemolyticus* ATCC 17802) 10^4^	vB_VpaS_OMN	MOI: 10^3^ surface direct application	1 log and 2 log reduction after 48 and 72 h of incubation, respectively	[124]
Oysters	*V. vulnificus* 10^6^	Phage pool (9 phages): S1, P3, P38, P53, P65, P68, P108, P111, P147	Unknown	5 log reduction after 18 h of incubation at 4 °C	[125]
Abalone flesh	*V. vulnificus* MO6-24/O 2 × 10^3^	VVP001	MOI: 10^5^ MOI: 10^6^	2.06 log reduction 2.51 log reduction	[126]

^1^ Content in food (CFU/g or mL, unless specified); * MOI (multiplicity of infection: ratio between bacteriophage and bacterial load).

### 3.2. Salmonella

Many *Salmonella* species have in common the ability to form biofilms, which are being considered as a factor to explain the extreme persistence of *Salmonella* in food-processing environments. Consequently, although the food industry has evolved in recent decades, the risk of contamination during food processing remains high. Due to the implication of *Salmonella* on FBO, the interest in phage biocontrol has increased in the last year as a new method of microbiological control applicable to food pathogens. In this regard, phages have been postulated as an alternative that could be applied directly to food or during food production as disinfectants, due to their stability under abiotic conditions, null toxicity, and selectivity in antimicrobial activity [127]. 

Different approaches (Table 3) have been used to assess phage success in controlling *Salmonella* biofilms in foodstuff [128,129]. Phages have also been applied to food as a natural preservative to treat chicken carcasses against *Salmonella* that is non-recoverable after phage application, resulting in the elimination of the pathogen [59,94]. In the same way, *Salmonella* contamination from broiler and turkey carcasses rinses was reduced by 100% and 60%, respectively [100]. In addition, a reduction of 2.0 logs of *S.* Enteritidis in packaged chicken breast after treatment with a cocktail of phages was observed, and a reduction of 0.9 logs was reached in egg samples after phage treatment [101]. Another work assessed the effect of one phage against *S.* Enteritidis on different matrices, such as eggs and chicken meat. After 12 h of treatment, reductions of 1.79 log CFU/mL and 1.83 log CFU/mL were achieved, respectively [102]. In breast samples, a reduction of 2 log CFU/mL in the *Salmonella* contamination was observed after the application of 2 different bacteriophages [103]. In addition, several commercial phages against *Salmonella* for the poultry industry are available, showing promising results in *Salmonella* biocontrol [104,105,106,107]. In one of the studied cases, phage treatment was the most effective, in comparison with peracetic acid and cetylpyridinium chloride, in controlling *Salmonella* in chicken breast fillets under room temperature conditions [104].

### 3.3. Listeria Monocytogenes

Despite the low incidence of listeriosis, its high fatality rate makes it the most frequent cause of foodborne infection-related deaths [28,130]. The main route of human infection is the consumption of contaminated food and, specially, ready-to-eat (RTE) food products that do not require further cooking between production and consumption [28]. The extraordinary capabilities of *L. monocytogenes* to survive and grow in a wide range of temperatures, pH levels, acidic solutions, and salt concentrations [131,132,133], as well as its ability to form biofilms [134,135], make it very challenging to remove from processing facilities, equipment, and environments [136]. 

Phage biocontrol shows great potential to be used as a safety control approach at the post-harvest stage of food production, in order to reduce the occurrence of *L. monocytogenes* in both the food-processing environment and the final food product (Table 3). Although few virulent *Listeria*-specific phages with potential for biocontrol have been characterized [137,138,139], some of them can infect not only the major *L. monocytogenes* serotypes, but also other species within the *Listeria* genus. 

Several studies have assessed the effectiveness of commercial products (Phage Guard Listex™ P100 by Micreos B.V., and ListShield™ by Intralytix) and other *Listeria*-specific phages to control this pathogen in contaminated food products, with variable success. Treatment effectiveness is mainly influenced by the MOI ratio, i.e., the ratio between phage dose and *Listeria* load. High concentration of phages allowing treatments at high MOI ratios ensure successful contact between phages and their hosts, leading to a more efficient reduction in *L. monocytogenes* on RTE chicken breast roll [111], dry cured ham [114], raw salmon [108], soft cheeses [117], and lettuce [110]. More successful treatments were observed when phage application occurred during or directly after product contamination [118] and under refrigerated post-treatment storage conditions [112,114,120]. 

It has been observed that *Listeria* reduction was more effective in fruit juices, where phages can diffuse until they meet their host, than in fruit slices, where phages are immobilized and cannot contact their hosts through limited diffusion [119]. Similarly, more important reductions were obtained in melon products (slices and juice; pH 5.8 ± 0.1) than in pear products (pH 4.7 ± 0.2), suggesting that pH could be also a key factor contributing to phage effectiveness [119]. These results indicated that, as suggested by other studies, food-related factors, such as physical form, pH, food composition, and/or the presence of specific compounds or substances, may interact with receptors or cell surfaces and interfere with phage diffusion, receptor recognition, and/or binding [115,140].

The intrinsic properties (e.g., lytic spectra, stability, etc.) of the different *Listeria*-specific phages directly affect treatment effectiveness. Better reduction was found on sliced apples after treatment with the cocktail Listshield [110] than with single phages [119], suggesting that the use of phage cocktails may contribute to better results [110]. Different reduction levels were also found after the application of different cocktails [118] and as a function of the target *L. monocytogenes* strain [115,141], underlying the importance of the lytic spectra of selected phages. Enhanced effectiveness of *Listeria*-specific phages has been reported when used in combination with other antimicrobials (e.g., bacteriocins or protective cultures) [108,109,112,113,116]. The application of phages as an innovative approach to eradicate *L. monocytogenes* biofilms in food processing environments and contact surfaces is another huge challenge that is currently being explored [120,140,142].

Overall, *Listeria*-specific bacteriophages and their cocktails could contribute, as an additional tool, to a multi-hurdle approach in order to safely reduce the occurrence and growth of *L. monocytogenes* in food products and food processing environments. 

### 3.4. Human Pathogenic Vibrio spp.

*Vibrio* spp. are natural hosts in marine waters, and, consequently, are also naturally present in seafood. *V. parahaemolyticus* constitutes the major causative agent for seafood-borne gastroenteritis by the consumption of contaminated products [81,121]. On the other hand, although less frequent, *V. vulnificus* is also an opportunistic foodborne pathogen that may cause lethal septicemia [125]. As was previously mentioned, *Vibrio* infections are being controlled as emerging foodborne agents worldwide, and AMR is also increasing. Consequently, the need for alternative pathogen-control tools has become an urgent necessity. As in the case of *Campylobacter*, there are no commercial solutions for controlling *Vibrio* spp. yet. However, in recent years, research into this kind of solution has increased, according to the emergence of *Vibrio* FBO. There are many works focused on the development and application of phages, especially on *V. parahaemolyticus* control (Table 3). 

For instance, the pVp-1 phage achieved a reduction of 6 log against a pandemic multidrug-resistant *V. parahaemolyticus* strain (CRS 09-17) when oysters were directly treated on their surfaces [80]. Other works have also reported an interesting effectivity when attempting to reduce *V. parahaemolyticus* counts in seafood products. Phage VPT02 showed about a 2 log drop in *V. parahaemolyticus* in raw fish flesh slices [121]. Similarly, the phages Vpp2 and OMN achieved reductions of about 2 logs in Manila clams and oysters, respectively [123,124]. Although more limited, the phages VPG01 and F23s1 have also demonstrated their capability, in solutions, to control the growth of *V. parahaemolyticus* in fresh fish and shrimps [81,122].

Regarding *V. vulnificus*, similarly, a phage cocktail has been also applied to reduce the load of *V. vulnificus* in eastern oysters from 10^6^ to 10^1^ CFU/mL [125]. A more recent study concluded that the VVP001 phage may be used to control *V. vulnificus* in a broad range of temperatures, ranging from −20 °C to 65 °C, showing a reduction of up to 2.51 logs of bacteria on abalone flesh [126].

These works have demonstrated that phages exhibit great potential as natural food preservatives for the biocontrol of potential *Vibrio* infections, as well as the prevention of contamination in diverse seafood-related circumstances, such as the storage and depuration steps of seafood [80] and the disinfection of seafood-processing equipment or utensils to prevent cross-contaminations [81].

## 4. Challenges of Using Phages for Food Safety

The use of phages as biocontrol tools has been gaining interest as a safety strategy in recent decades due to the emergence of AMR bacteria and the subsequent limited use of antibiotics in livestock and crops [143], thus remaining an interesting and natural alternative to combat bacteria. In terms of food safety, applications and advantages of phages have been already summarized in previous sections. However, although the results of the published studies appear to be promising, there are still some limitations that need to be addressed before their generalized use. To assist future phage-based real applications, pending issues and main challenges to be addressed shortly in future investigations are also reflected (Table 4).

The high specificity of phages, their ability to overcome resistance, and their self-dosage can be both strengths and weaknesses. Phage specificity is a major issue for their effectivity as antimicrobials in biocontrol. Host tropism is mostly dependent on receptors based in the cell walls or bacterial capsules. In this situation, building a collection of phages or biobanks to confront most of pathogenic bacteria strains could be a huge and time-consuming undertaking and, depending on the species, direct hunting could be both faster and costless. Interestingly, biobanks could allow ready-to-use phages to be available that can recognize and lyse a battery of bacteria. However, this requires performing phagograms to quickly select the potential phages to be used. This process, known as “phage matching”, could be easily performed with automated equipment, although is not common and the delay in determining the specific phage could be a problem. However, phage biocontrol can be effectively achieved as a customized treatment, which requires prior knowledge of the bacterial host and, most likely, phage hunting to select an efficient phage to control the target bacterium. Additionally, phages can be used as broad-range products by designing proper phage cocktails encompassing broad-range phages. Phage training (experimental evolution) or engineered phages could also help to broaden the host range and to obtain chimeric phages that could recognize multiple strains or species, although this may be detrimental to commensals. However, in food safety, and especially in the food industry, disinfectants to reduce bacterial burden are welcome, and phage-based products, including using phage-derived enzymes to eliminate bacterial biofilms, might be a promising solution as well. Indeed, phages encode several proteins with hydrolytic activity that can actively destroy the bacterial matrix composed of polysaccharide substances and can disrupt biofilms very effectively [144]. 

**Table 4:** Challenges and possible responses to resolve specific issues with using phages.

Challenge	Causes and/or Future Studies Needs	Refs.
Extreme host specificity	Personalized treatments Ready-to-use broad range products	[144,145]
Potential development of phage resistance	Formulations containing multiple phages (three or more, also known as phage cocktails) decrease the likelihood of phage resistance Combination with antibiotics (animals) or other preservatives (foods), could increase bacterial sensitivity through synergies Phage training to overcome resistance Genetic engineering	[145,146,147]
Phage stability and administration routes	Encapsulation Lyophilization Nanotechnology Study of pharmacokinetics and pharmacodynamics of phages Research on prophylactic uses of phages	[148,149,150,151]
Mobilization of resistant genes between bacteria	Lytic phages reduce transduction	[152,153]
Phage biobanking for immediate trials	Large collections of phages; public or private collections Phagograms Phage hunting could be the only solution in specific cases	[154]
Legislative approval	Ambiguous character of phages (non-living entities or pure biological macromolecular complexes) Agreed harmonized methods to verify effectivity and safety Evolving entities	[155,156]
Consumer acceptance	Unfounded fears and lack of contrastable information Need for public awareness (provide education on the safety, efficacy, and ubiquity of bacteriophages to stakeholders (processors, consumers, etc.)	[89]

Another drawback of some phages is that they might be intrinsically unstable; therefore, some phage-based products might require some procedures to be followed to maintain their stability and, thus, their infectivity. Embedding phages within a material, such as nanoparticles, has been proposed to control phage release and targeted delivery, and could be useful for long-term storage and provision of commercial products that could be stable at different conditions [148]. In addition, other preservation methods, such as freeze-drying, could be another option for long-term storage of phages; they are much cheaper, making them an interesting solution for the industry. However, some phages are not able to maintain infectivity after processing, and encapsulation could be the preferred solution for food protection [149,150]. In this context, it is important to study the pharmacokinetics and pharmacodynamics of phages in the environment and in animals, in order to ensure their stability and potential immune responses. Additional in vivo assays are required to ensure the safety and efficacy of the phage biocontrol. In this view, phage administration routes and procedures should be deeply investigated to determine the outcome of the therapy [151].

Another point to be addressed is that phages can mobilize genetic material encoding resistant genes between strains, thus promoting the spread of AMR, including to non-pathogenic bacteria. Although phages are evolving entities in nature, and this transfer could certainly occur in their natural environment, for biocontrol purposes, lytic and perfectly characterized (sequenced) phages are always preferred to reduce potential gene transfer [152]. In addition, detailed analysis of each phage genome must be performed, as it provides useful information for the selection of the most suitable phages. In addition, understanding phage–host interactions will be of special interest to anticipate potential failure treatments, such as the emergence of phage-resistant bacteria. Interestingly, phages can overcome resistance, adapting to the new environment faster than their hosts. In addition, phage cocktails can be a solution to reduce the emergence of phage resistance [153].

Finally, to be used, any phage application must be in compliance with legislation. Nevertheless, the great variability of phage morphologies and diversity, their intrinsic evolving, and their self-replication nature in the presence of the bacterial host create a challenge for regulatory agencies due to their intrinsic evolvability [155], and highlight the problem of subjecting all phage-derived products to the same regulation and procedures. As seen, legislation on the use of phages is a complicated issue and will delay commercialization and routine use of this promising virus. However, regulatory agencies should provide rapid guidance on phage biocontrol to address the emergence of resistant bacteria, since an alternative to antibiotics is necessary [157]. 

## 5. Conclusions

Although it is clear that no therapeutic or preventive treatment can or should replace good hygiene practices in food production, progressively, more studies have demonstrated that phage application can be a leading approach to controlling important foodborne diseases. Considering their natural properties and advantages, phages can be used at all stages of the agriculture supply chain to control microbial pathogens. They can be employed in every step, from agriculture (primary production) to biosanitization of food processing facilities and biopreservation of foodstuffs. Moreover, the aforementioned challenges are expected to be answered as the issue of AMR becomes more pressing. The creation of a legal framework to allow different applications of phages in reality, including in food safety, is an especially pressing issue.

## Figures and Tables

**Table 2:** Examples of the use of phages for controlling or reducing the incidence of different *Vibrio* spp. in aquaculture and closely related conditions.

Animal	Bacteria Load ^1^	Phage	Application Method and Dose ^2^	Bacterial Reduction	Ref.
Mussels	*V. parahaemolyticus,* naturally infected	Phage cocktail: VP10	Immersion ~10^3^	Undetectable levels in seawater, sediment, or mussels after 48 h	[79]
Oysters	*V. parahaemolyticus* CRS 09-17, AMR * 2.7 × 10^6^	pVp-1	Immersion 1.6 × 10^7^	Growth reduction >5 log after 72 h	[80]
Aquatic crustacean *Artemia franciscana*	*V. parahaemolyticus* FORC_023 10^4^	VPG01	Immersion 10^3^, 10^4^, 10^5^	2 log reduction Increased survival from 10% to 40% with higher phage concentration (10^4^ and 10^5^ PFU/mL).	[81]
Shrimps (*Penaeus monodon*) larvae	*V. harveyi* 10^5^	Bacteriophage of *V. harveyi*	Immersion 2 × 10^5^	3 log reduction in bacterial counts Increased larvae survival from 17% to 86% More effective than antibiotics (40% survival)	[82]
Gilthead seabream larvae	*V. harveyi* VH2 10^6^	Virtus	Immersion 10^7^	Increased survival of larvae: from <6% to >40%	[83]
Atlantic salmon	*V. anguillarum* PF4 5 × 10^5^	CHOED	Immersion (100L) 5 × 10^5^ Immersion (100L) 10^6^ Immersion (farm conditions) 5 × 10^7^	Increased survival from 5% to 70% after 10 d Increased survival from 5% to 100% after 10 d Increased survival from 65% to 100% after 9 d (protection up to 20 d)	[84]
Zebrafish larvae	*V. anguillarum* 10^6^	VP-2 phage	Immersion 10^8^	Increased survival from 83% to 98% after 72 h	[85]
Whiteleg shrimp larvae	*V. parahaemolyticus* ATCC 17802 2 × 10^6^	A3S Vpms1	Immersion 10^5^, 10^6^,10^7^	Increased survival of larvae from <60% to 80% (A3S phage), depending on the conditions	[86]
Shrimps (*Penaeus monodon*)	*V. harveyi*	Viha10 Viha8	Immersion 2 × 10^6^	Increased survival of larvae to >86% vs. ~65% survival with antibiotics	[87]

^1^ Water content (CFU/mL); ^2^ administered phage dose (PFU/mL); *AMR: antimicrobial-resistant strain.

## Data Availability

Not applicable.
